# Comment on Zieliński, G.; Gawda, P. Analysis of the Use of Sample Size and Effect Size Calculations in a Temporomandibular Disorders Randomised Controlled Trial—Short Narrative Review. *J. Pers. Med.* 2024, *14*, 655

**DOI:** 10.3390/jpm15030085

**Published:** 2025-02-26

**Authors:** Juliana Homem Padilha Spavieri, Thamiris Costa de Lima, Luiz Ricardo Garcêz, Roger Berg Rodrigues Pereira, Ana Carolina de Jacomo Claudio, Thais Cristina Chaves

**Affiliations:** 1 Graduate Program on Physical Therapy, Department of Physical Therapy, Federal University of São Carlos—UFSCar, São Carlos 13.565-905, SP, Brazil; jspavieri@estudante.ufscar.br (J.H.P.S.); thamiriscl@estudante.ufscar.br (T.C.d.L.); luizricardo@estudante.ufscar.br (L.R.G.); rogerpereira@estudante.ufscar.br (R.B.R.P.); anajacomo@estudante.ufscar.br (A.C.d.J.C.); 2 Department of Physical Therapy, Federal University of São Carlos—UFSCar, Rodovia Washington Luiz, Km 235, São Carlos 13.565-905, SP, Brazil

We have thoroughly reviewed the article titled “Analysis of the Use of Sample Size and Effect Size Calculations in a Temporomandibular Disorders Randomised Controlled Trial—Short Narrative Review” by Zieliński G and Gawda P, recently published in the Journal of Personalized Medicine (2024; 14: 655) [1]. We commend the authors on their insightful review and would like to contribute further to the discussion.

The authors highlight a significant trend in dental research publications and emphasize the critical importance of enhancing research quality. They underscore the pivotal role of statistical analysis, particularly in sample size calculation and effect size estimation, which are crucial for determining both statistical and clinical significance. Their short narrative review specifically analyzes these aspects in randomized controlled trials of temporomandibular disorders (TMDs).

We are writing to address concerns regarding Table 2 [1], which outlines the findings on the reporting of sample size calculations and effect size in the reviewed studies. We would like to draw attention to a specific point of the paper regarding the following study “Education-Enhanced Conventional Care versus Conventional Care Alone for Temporomandibular Disorders: A Randomized Controlled Trial” by Aguiar et al. (2024) [2], which received a score of 0 for sample size calculation. However, through a careful look at the methods section of the study conducted by Aguiar et al. it is possible to find a detailed description on how the sample size calculation was obtained. A protocol related to that study was published in the *Trials* journal, which also described how the sample size was calculated [3]. Hence, we respectfully request a retraction of the mistake related to the study of Aguiar et al. [2].

Additionally, the authors discussed the relationship between effect size estimation, statistical significance, and clinical application. The authors made a direct relation between the effect size and clinically meaningful changes. Nevertheless, the authors did not discuss that Cohen’s effect sizes, while widely used, pose challenges due to their arbitrary thresholds [4]. The terms “small”, “medium”, and “large” are relative, not only to each other but also to the area of behavioral science or even particularly to the specific field and research method being employed in any given investigation [5]. Moreover, alternative methods exist for assessing effect sizes in interventions, including the correlation between variables, regression coefficients, mean differences, or the risk of specific events occurring [6]. Unfortunately, these approaches were not reported as valid means to evaluate outcomes in clinical trials.

To assess the practical significance of a result, it is not enough to know the effect size [7]. Effect magnitude must be interpreted to extract meaning [7]. Effects by themselves are meaningless unless they can be contextualized against some frame of reference [7]. Whether an effect size should be interpreted as small, medium, or large depends on its substantive context and its operational definition [7]. Researchers should interpret the substantive significance of their results by grounding them in a meaningful context or by quantifying their contribution to knowledge, and Cohen’s effect size descriptions can be helpful but only as a starting point [7]. It is important to consider that an effect is influenced by when it occurs, where it occurs, and for whom it occurs [7].

Another important aspect to be considered is that to extract meaning from results, scientists need to look beyond *p* values and effect sizes and make informed judgments about what they see [7]. No one is better placed to do this than the researcher who collected and analyzed the data [8]. The fact that most published effect sizes go uninterpreted shows that many researchers are either unable or reluctant to take this final step [7]. Most are far more comfortable with the pseudo-objectivity of null hypothesis significance testing than with making subjective yet informed judgments about the meaning of results [7].

It has been highlighted that assessing the magnitude of reported differences in clinical trials also involves considering clinical relevance and patient impact [4]. Determining whether differences are clinically meaningful requires awareness of the context of current evidence [9] within the research field, which serve as a practical guide in evaluating study outcomes [4].

To enhance study quality, clarity in statistical analysis must be coupled with efforts to facilitate the clinical application of research findings [5]. This includes integrating probable effect sizes in a meaningful context, encompassing treatment costs, duration, expectations, and potential risks [4]. An interesting proposal that combines some of those elements to assess meaningful clinical effect is the smallest worthwhile effect which can be defined as the minimum benefit of an intervention that patients consider worthwhile given the costs, risks, and inconveniences [10,11].

We appreciate your attention to these issues, and we are confident that addressing these issues will undoubtedly benefit our scientific community.

Thank you for your consideration.
